# Experimental investigation of radiation shielding competence of B_2_O_3_-Na_2_O-Al_2_O_3_-BaO-CaO glass system

**DOI:** 10.1038/s41598-024-63329-9

**Published:** 2024-06-28

**Authors:** Esraa H. Abdel-Gawad, M. I. Sayyed, Taha. A. Hanafy, Mohamed Elsafi

**Affiliations:** 1 Environmental Studies Department, Institute of Graduate Studies and Research, Alexandria University, Alexandria, 21526 Egypt; 2 Department of Physics, Faculty of Science, Isra University, Amman, Jordan; 3 Department of Physics, Faculty of Science, University of Tabuk, Tabuk, Saudi Arabia; 4 Physics Department, Faculty of Science, Alexandria University, Alexandria, 21511 Egypt

**Keywords:** Attenuation parameters, Borate-based glasses, Radiation shielding, Glass shields, Ionizing radiation, Experimental nuclear physics, Theoretical nuclear physics

## Abstract

Aiming to extend the scope of utilizing glass in radiation shielding, this work investigates the radiation interaction response of a borate-based glass system. Four borate-glass samples of different substituting concentrations of calcium oxide ($70-x$)B_2_O_3_:$10$ Na_2_O $:5$ Al_2_O_3_ $:15$ BaO:$x$ CaO were prepared. To assess the shielding performance of the prepared glass samples, a high-purity germanium detector and different radioactive sources (different energies) were used. Via the narrow beam method, the linear attenuation coefficients (LACs) were experimentally measured. So, the transmission factor (TF), the half-value layer (HVL), the tenth value layer (TVL), the mean free path (MFP), and the radiation protection efficiency (RPE) were calculated for all prepared samples. It was observed that the increase of the concentration of calcium oxide in the proposed borate-based glass samples leads to improve their performance in shielding against radiation. At low energy, the RPE of the samples is almost 100%. However, it was observed that as energy of the radiation source increases, the shielding performance of the samples will decrease. High energy dependence was found when calculating TF, HVL, TVL, and MFP. They were increased with the increase of the energy of the incident photons. At 0.662 MeV, the TF values are equal to 79.26, 79.00, 79.72, and 78.43% for BNABC-1, BNABC-2, BNABC-3, and BNABC-4 in the same oder, respectively. The application of the proposed composition of borate-based glass as a transparent shield against low-energy ionizing radiation was highlighted.

## Introduction

Nowadays, scientists’ attention is attracted to develop new materials for conventional or novel applications. Glasses are becoming vital materials for innovative technological purposes. Various glass types are being utilized today, like borate, silicate, tellurite, and phosphate-based glasses. Lately, borate-based glasses are becoming progressively preferable for evolving glass applications thanks to the exceptional characteristics they have. Among the features of borate-based glasses which make them favorable, including high thermal stability and mechanical strength, the low melting and preparation temperature, make them simple to fabricate with reasonably low cost and easily tunable features compared to their other companions^1^. From previous studies^2–4^ it is obvious that borate-based glass characteristics are governed by the BO_4_, BO_3_, bridging, and nonbridging oxygen units. In a system made of borate-based glass, the vacancies created by the merging of three oxygen ions (O^−^) are filled with B^3+^ ions owing to the tiny size of the ions of boron, and this generates the units of BO_3_ (trigonal). The BO_3_ can create different secondary units when a modifier is introduced to the network of the glass. Thus, the triagonal units are considered the primary units of the borate-based glass structure^5^. The focus on borate-based glass systems is growing owing to their characteristics and their ability to accept different modifiers (metal oxides) which play diverse roles in fine-tuning borate glasses’ properties for optical, technical, and radiation protection applications^6–11^. For instance, research has shown that the presence of sodium oxide (Na_2_O) as a network modifier can enhance the mechanical strength, chemical stability, optical transparency, and electrical conductivity of borate-based glasses^12^ also it helps in stabilizing the glass so that its shielding properties against radiation is tested without difficulty^13^. Also, the presence of aluminum oxide can boost the properties of borate-based glasses as the ions of Al^3+^ join in the network of glass in the form of AlO_4_ and/or AlO_6_ structural units giving the glasses high resistance capability against radiation as a result of forming robust covalent bonds containing BO_4_ and AlO_4_ tetrahedral units^14,15^. Additionally, the presence of barium oxide (BaO) in the system of glass results in reinforcing the shielding capabilities of the glass network against radiation^16^. Moreover, the presence of calcium ions (Ca^2+^) with its attractive atomic absorption edge (K-edge) can augment the shielding capabilities of the glass systems. It can improve some properties of the borate-based glasses such as microhardness and chemical durability^17^. Thus, borate-based glasses are considered one of the efficient materials adopted for protection against radiation.

Many scientific researchers have investigated various borate-based glass systems. In the research of Alzahrani, J. and her team^18^, the parameters of shielding of borate-based glasses with Na_2_O content were estimated and analyzed. In their research work the interaction quantities of the glasses against neutron, photon, and charged radiation were determined using FLUKA Monte Carlo code, the calculator of XCOM, and theoretical relations. Besides the improved optical functions of their fabricated glass samples. They also exhibited beneficial radiation absorption performance, which recommends them for the roles of radiation absorption. Aboalatta et al.^19^ have used experimental and theoretical approaches to show that sodium-zinc-borate-based glasses with BaO content efficiently attenuate photons. The rise in BaO concentration boosted the shielding performance against gamma radiation of the fabricated samples of glasses. Also, the prepared glasses’ optical constants varied when the BaO concentration changed. Also, Aboud, and his research team have assessed the γ-radiation shielding characteristics of certain novel types of borate-based glasses made of $10$ BaO:$10$ Bi_2_O_3_:$10$ CdO:$x$ PbO:($70-x$)B_2_O_3_ ($0 \le x \le 20 mol\%$). Their results showed that the proposed composition of the glass is useful for developing improved shields to protect against ionizing radiation^20^. Singh, K. and his team^21^ started a helpful investigation to determine the values of the mass attenuation coefficient and related parameters against gamma-ray. Their glass systems were made of $x$ CaO:0.7B_2_O_3_:0.3–*x*)SrO and their data was helpful in establishing the potential uses of borate-based glass in the applications of gamma-ray shielding. Almuqrin, A. with her colleague Sayyed, M.^22^ theoretically studied the shielding characteristics of five borotellurite glasses containing calcium oxide by Phy-X/PSD. The mass attenuation coefficient was found to change as the concentration of the modifiers inside the glass network changed.

The literature review has prompted us to extend the ongoing research on promising features of borate-based glasses. Accordingly, this study also aims to extend the scope of utilizing glass shields to protect against radiation. The goal of this novel study is to estimate the radiation-interaction response parameters of ($70-x$)B_2_O_3_:$10$ Na_2_O $:5$ Al_2_O_3_ $:15$ BaO:$x$ CaO ($x=5$, $10$, $15,$ and $20$ $mol\%$) glasses in a wide range of energies. The findings of this research will be beneficial as an added value to the borate-based glass literature as it highlights their applications as transparent shields against radiation.

## Experimental work

### Samples preparation

**Figure 1:**
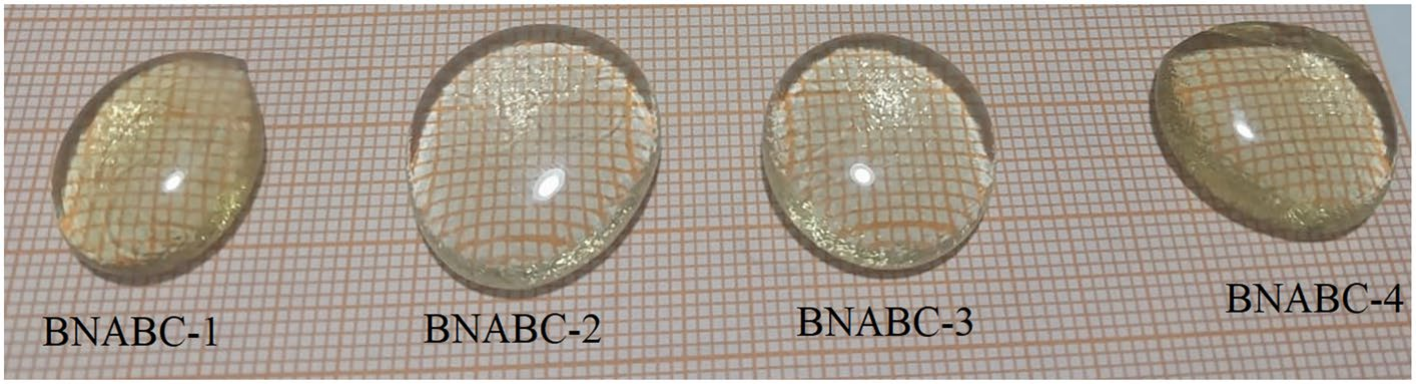
Prepared glass samples.

**Figure 2:**
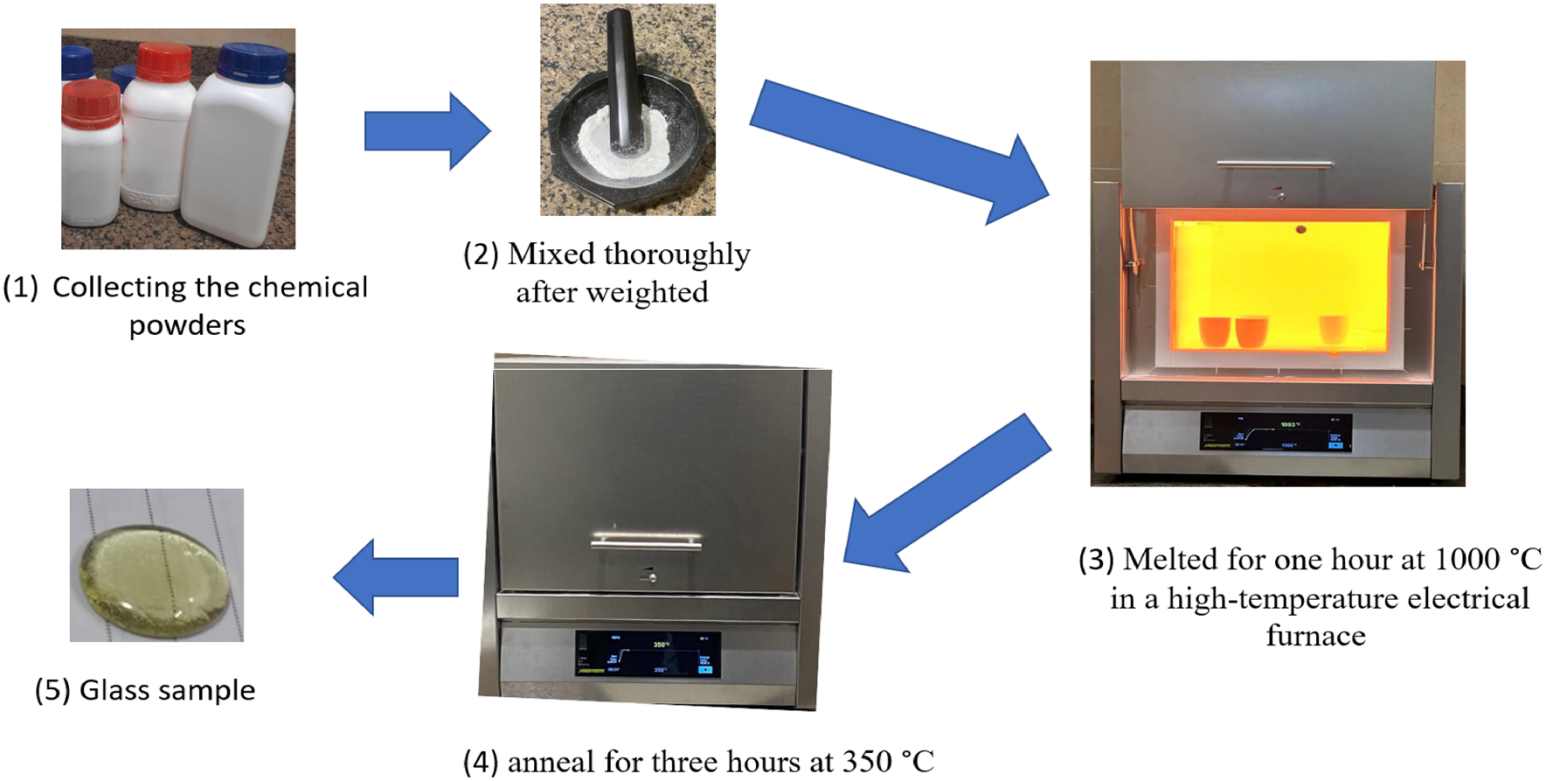
Preparation steps.

Borate-based-glass samples were prepared via the melt quenching method^23,24^ with fixed amounts of sodium oxide (Na_2_O), aluminum oxide (Al_2_O_3_), and barium oxide (BaO), and different concentrations of calcium oxide (CaO) which were selected to gradually replace boron oxide (B_2_O_3_) in the prepared samples. A total of four glass samples were prepared via chemical powders of the metals oxides ($70-x$)B_2_O_3_:$10$ Na_2_O $:5$ Al_2_O_3_ $:15$ BaO:$x$ CaO ($x=5$, $10$, $15,$ and $20$ $mol\%$). After weighing the required chemicals, a batch of $20$ grams of the glass powder was mixed completely. Then the sample was contained in a high-purity alumina crucible to enter an electrical furnace for $2$ hours at $1100^\circ C$. In another furnace, glass annealing for $3.5$ hours at $400^\circ C$ were carried out to eliminate internal stresses. For convenience, the samples were labeled as “BNABC-1” ($x= 5 mol\%$), “BNABC-2” ($x =10 mol\%$), “BNABC-3” ($x = 15 mol\%$), and “BNABC-4” ($x = 20 mol\%$), with Table 1 summarizing the glasses' composition. The density of the glasses was calculated using Archimedes principle. Figure 1 shows a real picture of the prepared samples and Fig. 2 displays the glass samples preparation steps.

**Table 1:** Chemical composition of the prepared glass samples.

Glass sample code	Glass sample composition ($mol\%$)					Density ($g/{cm}^{3}$)
	B_2_O_3_	Na_2_O	Al_2_O_3_	BaO	CaO	
BNABC-1	$65$	$10$	$5$	$15$	$5$	$3.053$± 0.008
BNABC-2	$60$	$10$	$5$	$15$	$10$	$3.094\pm 0.011$
BNABC-3	$55$	$10$	$5$	$15$	$15$	$3.137\pm 0.011$
BNABC-4	$50$	$10$	$5$	$15$	$20$	$3.181\pm 0.009$

### Measurements of radiation shielding parameters

**Figure 3:**
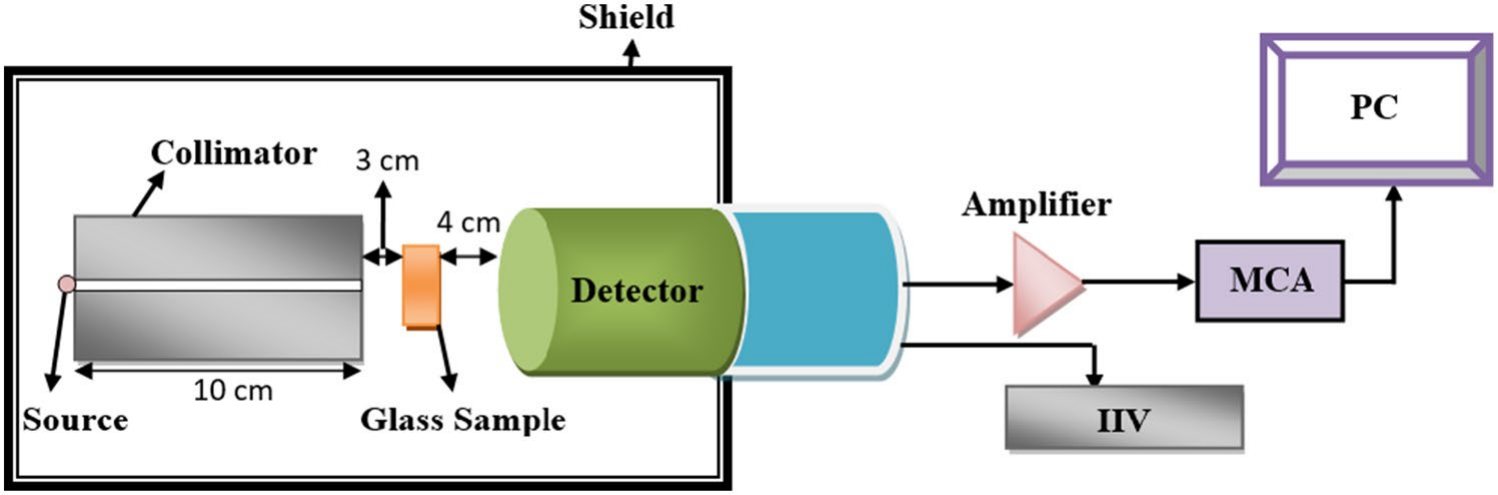
Experimental setup of measuring the shielding parameters.

To measure the shielding parameters of the aforementioned glass samples, a high-purity germanium (HPGe) detector and different radioactive sources (^60^Co, ^137^Cs, and ^241^Am) were used and their activities and characteristics were reported in the literature^25–27^. Firstly, ^60^Co and ^137^Cs point sources were used to calibrate the detector’s efficiency and energy. The experimental setup of the present research is illustrated in Fig. 3. The measurements depended on the narrow beam technique in which the glass samples, each in turn, were placed between the detector and the radioactive source which emitted photons of a certain energy (the lead collimator with iner diameter 8 mm and length10 cm was used to achieve narrow beam). The absorbing glass material attenuated the incident photons to a specific degree based on the composition of each sample. During a particular time, the peaks associated with the incoming energy photons are formed. The area under these peaks can be calculated using Genie-2000 software, and the count rate (area per time) represents the intensity of the incoming photon.

### Radiation attenuation coefficients

A gamma photon is attenuated as it is transmitted within a certain thickness of a material. Higher attenuation for a particular thickness indicates an extra effective shielding material. Therefore, choosing the material of shielding is governed by the degree of attenuation which is given by Lambert–Beer law^28^:

1 $$I={I}_{0 }{e}^{-\mu x}$$

where $\mu$ (${cm}^{-1}$) is the linear attenuation coefficient or LAC, $I$ is the intensity of an attenuated photon, ${I}_{0}$ is the intensity of an incident photon, and $x$ is the material thickness.

Other crucial factors that can be calculated from $\mu$ are the transmission factor (TF) which measures unabsorbed radiation , half-value layer (HVL), tenth value layer (TVL), mean free path (MFP), and the radiation protection efficiency (RPE). They can be determined via the following relations:

2 $$TF = \,\frac{I}{{I_{o} }} = e^{( - \mu x)}$$

3 $$HVL=\text{ln}\left(2\right)/ \mu$$

4 $$TVL=\frac{Ln 10}{LAC}$$

5 $$MFP=1/\mu$$

6 $$RPE\%=(1-\frac{I}{{I}_{0}})\times 100$$

## Results and discussion

**Figure 4:**
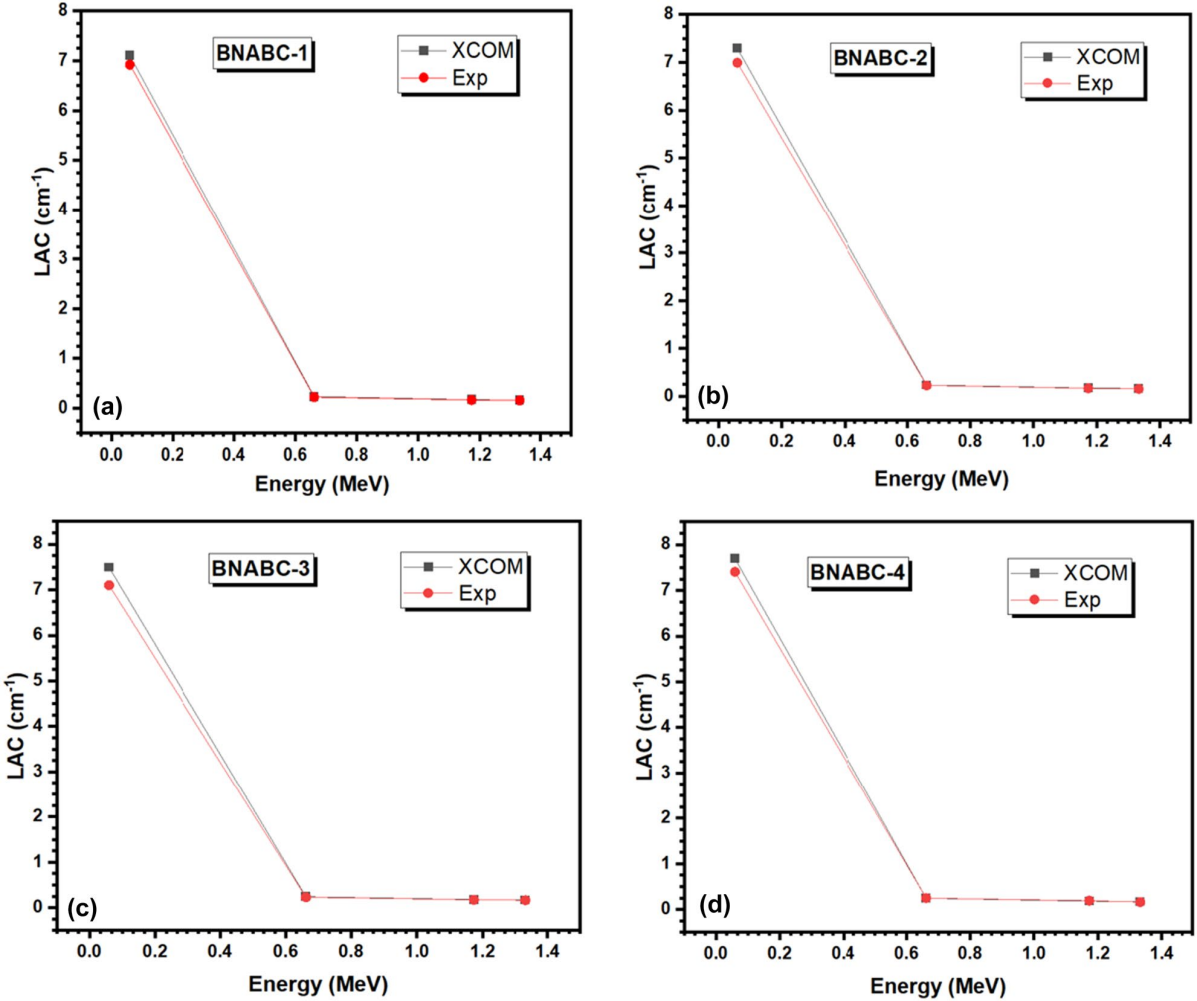
(**a**–**d**): The experimentally determined LAC values for the four BNABC-X samples against their corresponding theoretical values.

Figure 4a–d shows a comparison between the experimental and the theoritcal values of LAC, for the four BNABC-X samples. Between the four samples, BNABC-3 sample at 0.060 MeV exhibits the highest deviation between XCOM and experimental values, where XCOM calculates LAC of 7.490 ± 0.032 cm^−1^, while the experimentally measured value is 7.0983 ± 0.042 cm^-1^ (5.52% deviation). At other energies and for other samples, such as at 1.173 MeV, the deviation is smaller; in this example, it is equal to 3.45%, or 0.172 ± 0.011 cm^−1^ for XCOM and 0.1661 ± 0.009 cm^−1^ for the experimental method. The percentage deviation is within 5% for most of the samples and energies. Therefore, the calculated difference is well within the acceptable range. This means that these values can confidently be used to determine other radiation shielding parameters.

**Figure 5:**
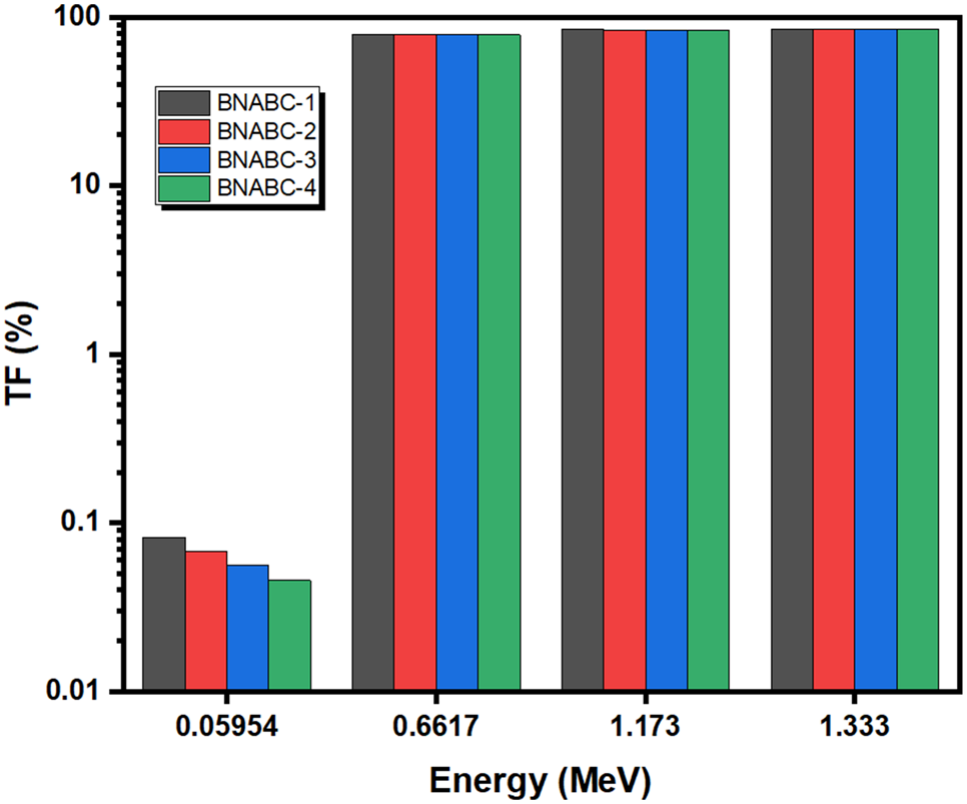
The transmission factor for the BNABC-X samples.

The TF, or transmission factor of a material, measures the percentage of photons that penetrate through the sample at a certain energy and thickness for the material. Figure 5 shows the TF values for the investigated samples, with thikness of 1 cm, at the four definit energies. When attenuating photons with an energy of 0.060 MeV, the TF values are extremely low, between 0.05 and 0.08%. This means that an overwhelming majority of radiation at this energy is absorbed by 1 cm thick samples of this type of the glass system. With the increase of the energy of the incident photons to 0.662 MeV and more, the TF values will be increased. At 0.662 MeV, the TF values are equal to 79.26, 79.00, 79.72, and 78.43% for BNABC-1, BNABC-2, BNABC-3, and BNABC-4 in the same order, respectively. However, if the photons energy is equal of 1.173 MeV, the TF takes values ranged from 83.57% to 84.21%. Moreover, if the absorbing photons energy becomes 1.333 MeV the values of TF take the values range from 84.55% to 85.15%. Therefore, the higher the energy of the incoming photons, the greater the TF values for the samples are.

**Figure 6:**
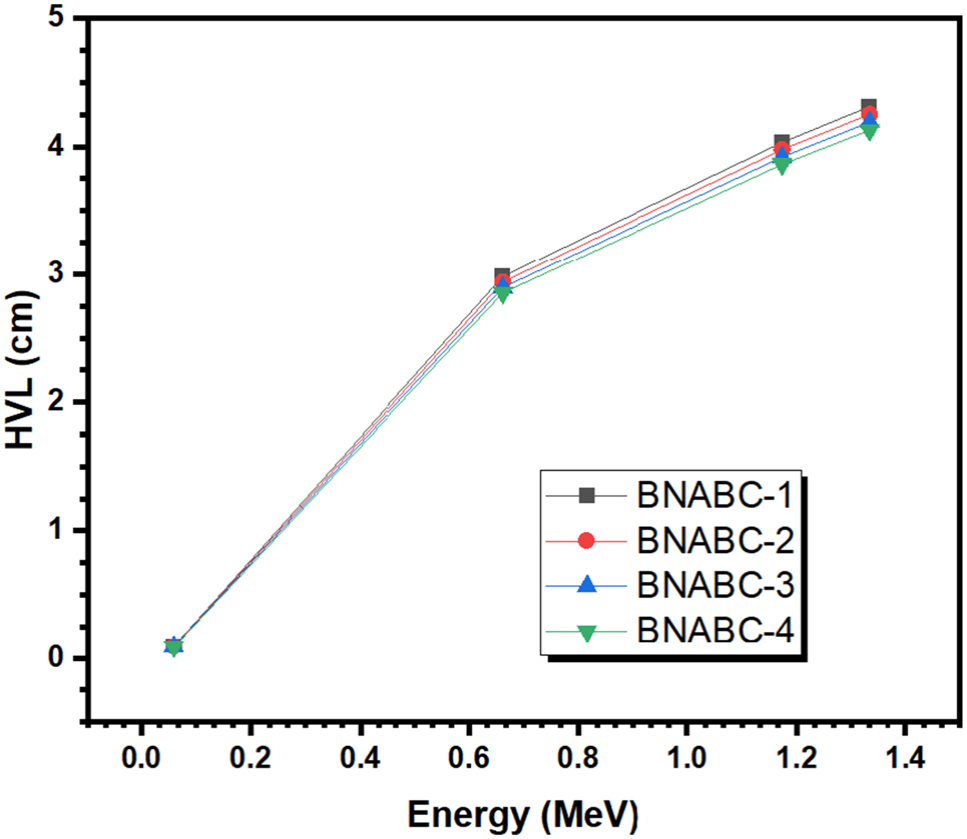
The half value layer of the BNABC-X samples.

HVL is a measure of how thick a material needs to be to attenuate half of the total incoming photons. The HVLs of the BNABC-X samples are demonstrated in Fig. 6 as a function of the energy of the incoming photons. The BNABC-1 sample has the highest HVL at all tested energies, followed by the BNABC-2 sample, BNABC-3 sample, and BNABC-4 sample. For example, at 0.662 MeV, the HVL values are equal to 2.983, 2.940, 2.896, and 2.853 cm for BNABC-1, BNABC-2, BNABC-3, and BNABC-4 in the same order, respectively, while at 1.333 MeV they are respectively equal to 4.313, 4.253, 4.192, and 4.130 cm. Therefore, the BNABC-4 sample has the lowest HVL for four tested glasses at all energies of incident photons, while the BNABC-1 sample has the largest HVL. The BNABC-4 sample has the highest CaO content and lowest B_2_O_3_ concentration of the four glasses. This means that higher CaO content, and lower B_2_O_3_ content, correlate with a smaller glass thickness being required to attenuate the same number of photons.

**Figure 7:**
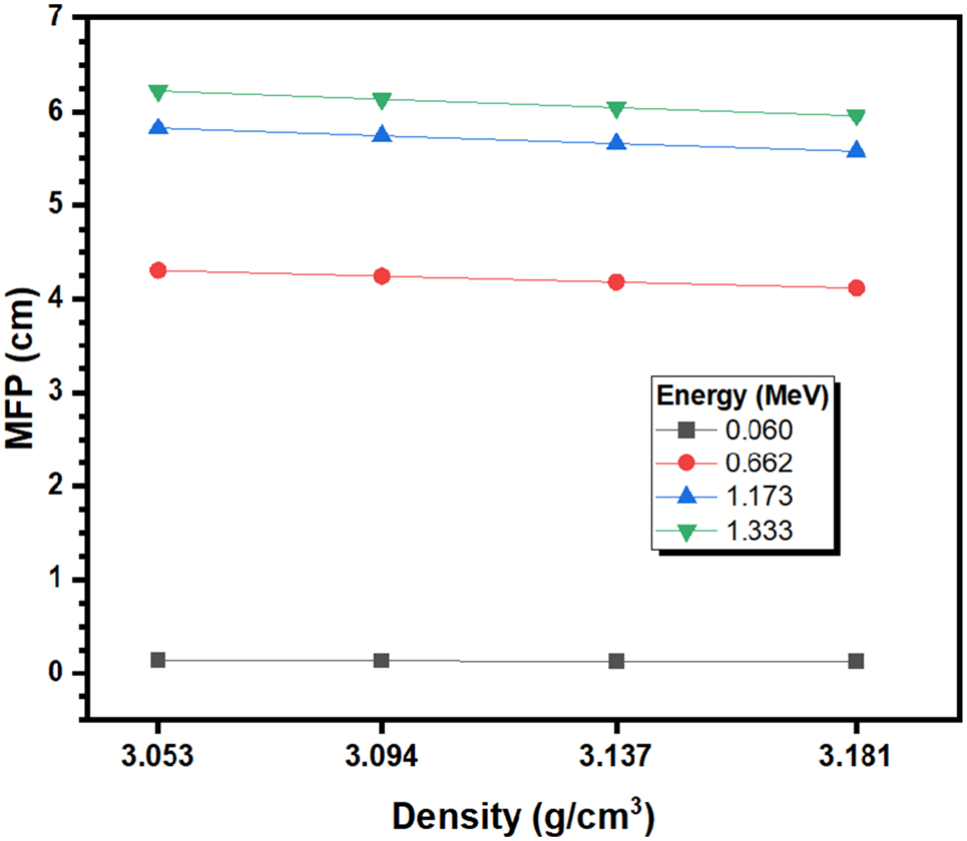
The mean free path of the BNABC-X glass samples as a function of their density.

Figure 7 demonstrates the MFP of the tested BNABC-X glass samples as a function of their density at several tested energies of the incident photons. The figure reveals that there is an increasing trend between MFP and energy, with the samples having the lowest MFP at 0.060 MeV, the lowest tested energy, and the highest MFP at 1.333 MeV, the highest tested energy. For instance, for glass with a density of 3.053 g/cm^3^, the MFP values are equal to 0.141, 4.303, 5.820, and 6.223 cm at 0.060, 0.662, 1.173, and 1.333 MeV, respectively. Moreover, at a density of 3.181 g/cm^3^, the MFP values at the same respective energies are equal to 0.130, 4.116, 5.573, and 5.959 cm. Therefore, photons with a higher energy have an easier time passing through the BNABC-X glasses, leading to fewer collisions and a higher MFP. The figure also shows that by the increase of the density of the samples, the MFP values are reduced. For example, at 0.662 MeV, the MFP values are equal to 4.303, 4.241, 4.179, and 4.116 cm at densities of 3.053, 3.094, 3.137, and 3.181 g/cm^3^, respectively, while at 1.333 MeV, the MFP values at the same respective densities are equal to 6.223, 6.136, 6.047, and 5.959 cm. Thus, by the increase of the density of the samples, more collisions occur within the samples, leading to greater attenuation. Since replacing the B_2_O_3_ in the samples with CaO leads to an increase in density, glass samples with greater CaO content and less B_2_O_3_ content have more desirable shielding properties.

**Figure 8:**
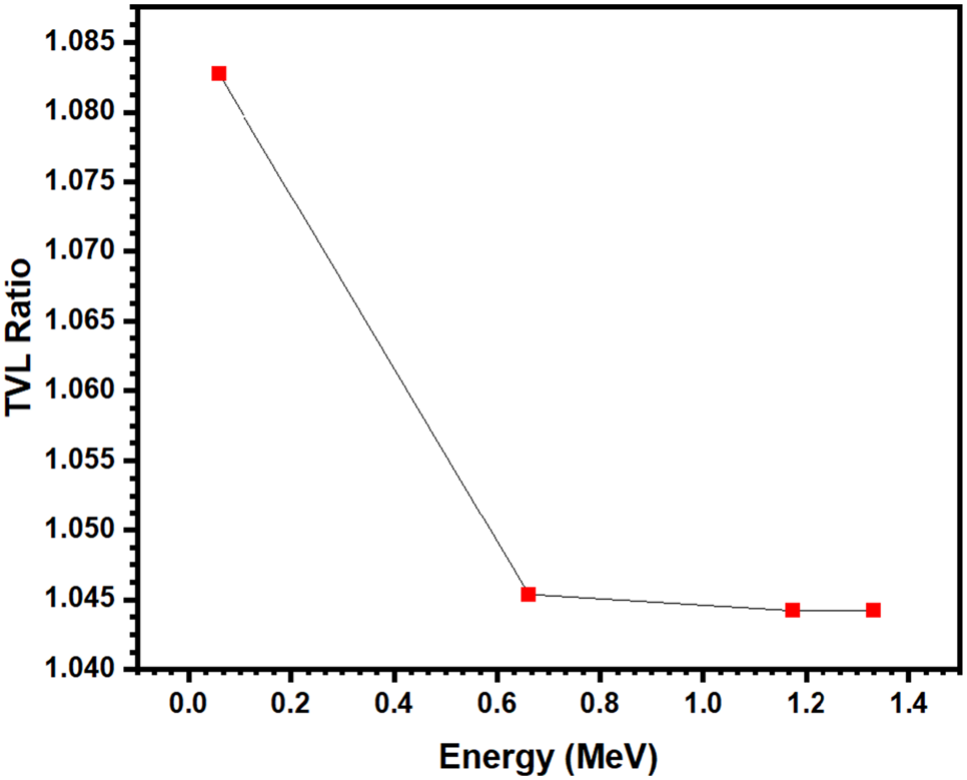
the TVL ratio between the BNABC-1 sample and the BNABC-4 sample.

Figure 8 demonstrates the TVL ratio between the BNABC-1 sample and the BNABC-4 sample at the four tested energies. The TVL ratio starts at 1.0827 at 0.060 MeV, and drops to 1.0454 at 0.662 MeV, and then flattens out to 1.0442 at 1.173 and 1.333 MeV. These results show that there is a greater difference in TVL values at lower energies compared to higher energies. Additionally, the TVL ratio is greater than one at all four of these energies, which means that BNABC-1’s TVL is greater than BNABC-4’s TVL across these energies. Therefore, the BNABC-4 sample, which has greater CaO content and a higher density than the BNABC-1 sample, exhibits more desirable shielding ability, especially against low-energy photons.

**Figure 9:**
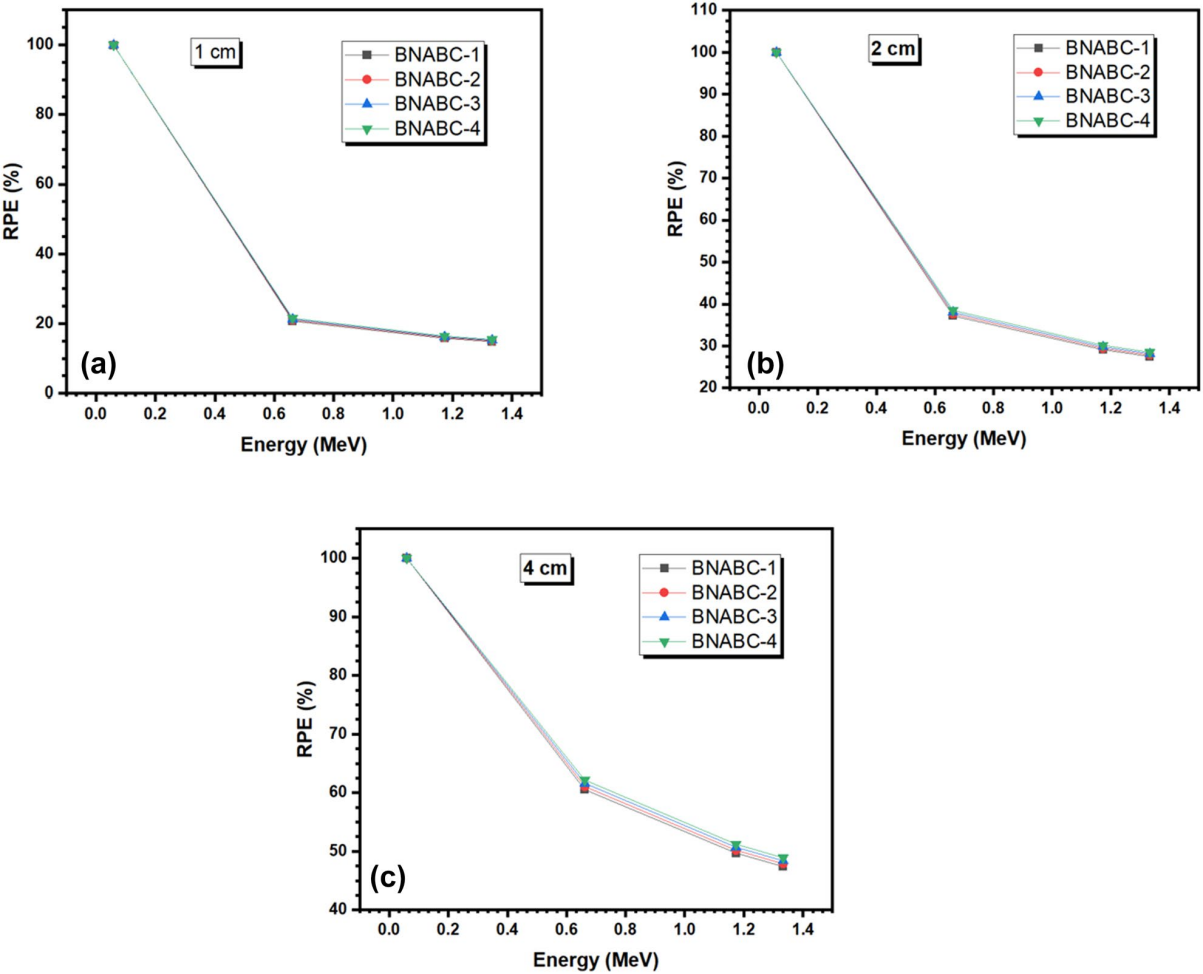
(**a**–**c**) The radiation shielding protection, or RPE, of the tested samples with a thickness of 1 cm, 2 cm, and 4 cm respectively.

The radiation shielding protection, or RPE, of the tested samples with a thickness of 1 cm, 2 cm, and 4 cm are shown in Fig. 9a–c at the four tested energies. Focusing on the samples at a thickness of 1 cm, it is clear that at low energy photons, 0.060 MeV, the RPE of the samples is almost 100% (99.92–99.95%). However, at higher energy photons, the RPE will decrease. Specifically, the RPE is between 20.74 and 21.57% at 0.662 MeV, between 15.79 and 16.43% at 1.173 MeV, and 14.85 and 15.45% at 1.333 MeV. The higher energy photons lead to a lower protection efficiency for all four glasses. The figure also shows that the BNABC-4 sample has the highest RPE at all energies, while the BNABC-1 sample has the lowest RPE at all energies. Analyzing the RPE values at an energy of 0.662 MeV and a thickness of 2 cm, the RPE values are equal to 37.17, 37.60, 38.04, and 38.48% for BNABC-1, BNABC-2, BNABC-3, and BNABC-4 in the same order, respectively. Meanwhile, at an energy of 1.173 MeV and a thickness of 4 cm, the RPE values are respectively equal to 47.42%, 47.90%, 48.39%, and 48.89%. These values demonstrate that the greater the CaO content in the glass system, the greater the radiation protection efficiency that a glass within this glass system has. Additionally, by the increase of the thickness of the shield also will increase the RPE of the glasses. For example, for the BNABC-3 glass at 1.173 MeV, its RPE values at a thickness of 1, 2, and 4 cm are 16.21%, 29.79%, and 50.70% in the same order, respectively. Therefore, the thickness of the glasses should be maximized within the constraints of the application to gain the greater possible attenuation.

## Conclusion

The present work is an experimental investigation of a promising borate-based glass that contains different contents of calcium oxide (CaO). Depending on the composition of each glass sample ($70-x$)B_2_O_3_:$10$ Na_2_O $:5$ Al_2_O_3_ $:15$ BaO:$x$ CaO ($x=5$, $10$, $15,$ and $20$ $mol\%$), the absorbing glass material attenuated the incident photons to a different extent. The density of the prepared four glass samples was increased going from sample BNABC-1 to sample BNABC-4 owing to the increase in CaO content which substituted the BaO content gradually. The shielding parameters like transmission factor (TF), half-value layer (HVL), tenth value layer (TVL), mean free path (MFP), and the radiation protection efficiency (RPE) were calculated using LAC. It was revealed that the BNABC-4 sample, which has greater CaO content, exhibits more desirable shielding ability, especially against low-energy photons as it exhibits almost 100% protection from radiation. BNABC-4 sample blocks more photons from penetrating from the sample (lowest TF). This is due to the createation of more collisions of photons within the sample (lowest MFP), and offers the least thickness to protect against radiation (HVL & TVL) through all photon energies. For low-energy of the incident photons, the values of TF, HVL, TVL, and MFP were extremely low. But their values start to rise when the incoming photons' energy exceeds 0.662 MeV for all the tested samples. Conclusively, this type of borate-based glass composite offers a valuable and suitable shielding material against low radiation level sources. In order to fully comprehend the properties of glass composites as shielding materials, future research should look into incorporating other metal oxides into different kinds of glass, such as tellurite, borate, and silicate-based glasses.

## Data Availability

The data that support the findings of this study are available from the corresponding author upon reasonable request.
